# TRF1 and TRF2: pioneering targets in telomere-based cancer therapy

**DOI:** 10.1007/s00432-024-05867-3

**Published:** 2024-07-16

**Authors:** Anoop Kallingal, Radosław Krzemieniecki, Natalia Maciejewska, Wioletta Brankiewicz-Kopcińska, Maciej Baginski

**Affiliations:** 1 Department of Pharmaceutical Technology and Biochemistry, Faculty of Chemistry, Gdansk University of Technology, Gdansk, 80-233 Poland; 2 Department of Medical Genetics, Institute of Clinical Medicine, University of Oslo, Oslo, Norway

**Keywords:** Cancer, Shelterin complex, TRF1, TRF2

## Abstract

This article presents an in-depth exploration of the roles of Telomere Repeat-binding Factors 1 and 2 (TRF1 and TRF2), and the shelterin complex, in the context of cancer biology. It emphasizes their emerging significance as potential biomarkers and targets for therapeutic intervention. Central to the shelterin complex, TRF1 and TRF2 are crucial in maintaining telomere integrity and genomic stability, their dysregulation often being a hallmark of cancerous cells. The article delves into the diagnostic and prognostic capabilities of TRF1 and TRF2 across various cancer types, highlighting their sensitivity and specificity. Furthermore, it reviews current strides in drug discovery targeting the shelterin complex, detailing specific compounds and their modes of action. The review candidly addresses the challenges in developing therapies aimed at the shelterin complex, including drug resistance, off-target effects, and issues in drug delivery. By synthesizing recent research findings, the article sheds light on the intricate relationship between telomere biology and cancer development. It underscores the urgency for continued research to navigate the existing challenges and fully leverage the therapeutic potential of TRF1, TRF2, and the shelterin complex in the realm of cancer treatment.

## Introduction

The development of new cancer drugs targeting specific cellular components has become increasingly important in the fight against cancer. Among these targets, telomeres and their associated proteins, particularly the shelterin complex, have gained significant attention. This article dives into the need for new cancer drugs acting on telomeres, providing a basic understanding of the shelterin complex, and focusing on two of its critical proteins: TRF1 and TRF2. Telomeres, the protective caps at the ends of eukaryotic chromosomes, play a crucial role in maintaining genomic stability. They consist of repetitive DNA sequences and are safeguarded by a specialized protein complex known as shelterin. This complex protects telomeres from being recognized as DNA damage sites, thereby preventing inappropriate repair activities that could lead to genomic instability, a characteristic of cancer cells (Blackburn 2001a; Lange 2005a). The need for new cancer drugs becomes clear when considering the unique characteristics of cancer cells, such as their ability to maintain telomere length, which is often facilitated by the enzyme telomerase. This ability allows cancer cells to bypass the normal limits of cellular lifespan, continuing to divide indefinitely (Shay and Wright 2019a). The shelterin complex is composed of six proteins: TRF1, TRF2, POT1, TIN2, TPP1, and RAP1 (Hu et al. 2023). TRF1 and TRF2 (Telomeric Repeat-binding Factor 1 and 2) are particularly significant due to their direct binding to the double-stranded telomeric DNA. TRF1 is involved in telomere length regulation and has been shown to negatively regulate telomerase activity by fine tuning the telomere length by different mechanisms (Oh et al. 2021; Wang and Wu 2021). Mutations or alterations in TRF1 expression are associated with various cancer types, suggesting its potential as a therapeutic target (van Steensel and de Lange 1997a; Martínez and Blasco 2017). TRF2 is critical for protecting telomeres from activating DNA damage responses. It plays a crucial role in maintaining the telomere loop (T-loop) structure, by fusing the single-strand of DNA with its double-strand component. Disruption of TRF2 leads to telomere uncapping, triggering DNA damage responses and potentially leading to cell death or senescence (Karlseder et al. 1999a; Imran et al. 2021). This characteristic makes TRF2 another promising target for cancer therapy, as its inhibition could induce a crisis in cancer cells with short telomeres. The biological role of the shelterin complex, particularly TRF1 and TRF2, extends beyond just protecting telomere ends. It is intricately involved in the process of cell aging and senescence. Cellular senescence is a state of permanent growth arrest that acts as a barrier to tumorigenesis. However, as telomeres shorten with each cell division, cells eventually reach a critical length that triggers senescence (Harley et al. 1990). Cancer cells often bypass this mechanism, one of the causes may be an altered function of shelterin components, which allows them to maintain telomere length and continue proliferating (Shay and Wright 2019b). In the context of cancer, the roles of TRF1 and TRF2 become even more critical. Some cancer types exhibit upregulation of these proteins, contributing to telomere maintenance and the immortalization of cancer cells. This observation has led to the hypothesis that targeting TRF1 and TRF2 could be a viable strategy for cancer therapy. By disrupting the normal function of these proteins, it may be possible to induce telomere dysfunction and, consequently, cell death in cancer cells (Biroccio et al. 2013).

### Sheltrin complex in cancer development

**Fig. 1:**
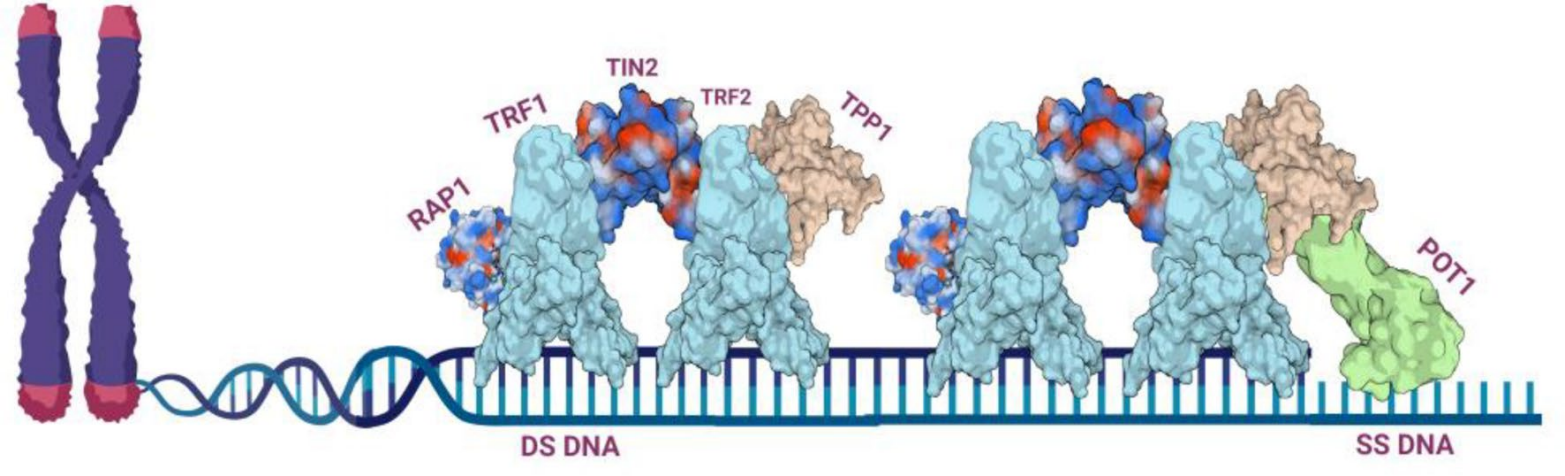
Schematic representation illustrating the structural arrangement of the sheltrin complex on the chromosomal end. The assembly, comprised of RAP1, TRF1, TRF2, TIN2, and TPP1, facilitates the binding to double-stranded DNA, while the interaction with POT1 within the complex aids in the binding to single-stranded DNA

The shelterin complex, a critical entity in telomere biology, plays a pivotal role in cancer development. This complex is composed of six core proteins: TRF1, TRF2, RAP1, TIN2, TPP1, and POT1, each of which contributes uniquely to telomere maintenance and thus influences cancer progression Fig. 1. The intricate structural organization of these proteins and their collaborative functioning are essential in maintaining chromosomal stability, a key factor in preventing oncogenesis. TRF1 and TRF2 bind directly to double-stranded telomeric DNA. TRF1 is crucial for telomere length regulation and helps in telomere replication. Studies have shown that TRF1’s overexpression or downregulation can lead to telomere length dysregulation, a common feature in cancerous cells (Steensel et al. 1998; Khodadadi et al. 2021). TRF2, on the other hand, is vital for protecting telomeres from being recognized as sites of DNA damage. It forms a protective cap at telomere ends, and its overexpression in cancer cells helps maintain telomere integrity, allowing for continued proliferation despite critically short telomeres (Karlseder et al. 1999a; Kumar et al. 2022). RAP1, recruited to telomeres by TRF2, plays a supplementary role in telomere protection. It has been implicated in the regulation of telomere length and the prevention of non-homologous end joining (NHEJ), a key factor in genomic stability (Martínez et al. 2009). The role of RAP1 in cancer is less direct but is thought to modulate TRF2’s functions in telomere capping and protection. TIN2 acts as a central hub within the shelterin complex, bridging the interactions between TRF1, TRF2, and TPP1/POT1 subcomplexes. Its role in cancer involves the regulation of telomere length and the stabilization of the shelterin complex (Kim et al. 1999a; Fan et al. 2021). TIN2’s dysfunction can disrupt shelterin’s protective functions, leading to telomere instability and potentially contributing to oncogenic processes. TPP1 and POT1 together form a subcomplex that binds to the single-stranded overhang of telomeric DNA. POT1 is crucial for regulating telomerase activity and protecting telomere ends from eliciting a DNA damage response. TPP1 aids in POT1 recruitment and also interacts with telomerase, playing a role in telomere length regulation. The dysregulation of TPP1/POT1 can lead to either aberrant telomere elongation or excessive shortening, both of which are implicated in cancer development (Hockemeyer et al. 2005a). The combined actions of these proteins within the shelterin complex are crucial for telomere maintenance and genome integrity. In cancer, alterations in any component of the shelterin complex can lead to telomere dysfunction, resulting in genomic instability, a hallmark of cancer cells. For instance, mutations or altered expression levels of shelterin components have been observed in various cancers, correlating with telomere length abnormalities and increased genomic instability (Lange 2005b). The shelterin complex interacts with various signaling pathways and proteins involved in the DNA damage response (DDR). This interaction is crucial in preventing inappropriate DDR activation at telomeres, which can lead to cell cycle arrest or apoptosis. In cancer cells, these interactions are often dysregulated, leading to inappropriate telomere maintenance and contributing to the genomic instability that drives cancer progression (Palm and de Lange 2008). Research into targeting the shelterin complex in cancer therapy is gaining momentum. Given its central role in maintaining telomere integrity and chromosomal stability, disrupting shelterin-telomere interactions presents a potential strategy for selectively targeting cancer cells with altered telomere maintenance mechanisms (Martínez and Blasco 2011).

### TRF1 and TRF2 in telomere biology and chromosome stability

Telomere Repeat-binding Factors 1 and 2 (TRF1 and TRF2) are crucial components in the field of telomere biology and chromosome stability. These proteins, essential to the shelterin complex, play crucial roles in maintaining telomere length and structure, thereby contributing significantly to chromosome stability and cellular lifespan. Their importance is emphasized by researchers indicating that dysfunctions in telomere maintenance are associated with aging, cancer, and other diseases. TRF1 primarily functions in the regulation of telomere length. It binds to double-stranded telomeric DNA and negatively regulates telomere length by controlling the access of telomerase, an enzyme responsible for adding telomeric repeats to the ends of chromosomes, to the telomere (van Steensel et al. 1998; Khodadadi et al. 2021). This regulation ensures that telomeres do not become excessively long, which is crucial for maintaining genomic integrity. In addition, TRF1 is involved in telomere replication. It facilitates the replication of telomeric DNA by unwinding t-loop structures and recruiting replication proteins to telomeres (Sfeir et al. 2009). TRF2, on the other hand, plays a critical role in protecting telomere ends from being recognized as sites of DNA damage. It stabilizes the t-loop structure at the end of telomeres, thereby preventing activation of DNA damage response pathways (Lange 2005b). This function of TRF2 is essential for preventing end-to-end chromosome fusions, a phenomenon that leads to genomic instability and is a hallmark of cancer cells (Celli and de Lange 2005). Moreover, TRF2 has been implicated in the regulation of telomere length, although its role is less direct compared to TRF1 (van Steensel et al. 1998; Fan et al. 2021). The synergy between TRF1 and TRF2 in maintaining telomere structure and function is of great significance. While TRF1 ensures the correct length and replication of telomeres, TRF2 protects the ends of chromosomes from damage. This dual action is critical for chromosome stability. When telomeres are too short, they can trigger a DNA damage response, leading to cell cycle arrest or apoptosis. Conversely, overly long telomeres can lead to increased recombination and genomic instability (Blackburn 2001b). In terms of cellular lifespan, TRF1 and TRF2 have profound implications. Their roles in regulating telomere length and protecting telomere integrity are linked to the cellular aging process. Telomere shortening is a hallmark of cellular aging, and the ability of TRF1 and TRF2 to modulate telomere length and structure influences the replicative capacity of cells. Cells with dysfunctional TRF1 or TRF2 exhibit premature senescence, indicating a direct link between telomere maintenance and cellular lifespan (Smogorzewska et al. 2000).

### TRF1 and TRF2 dysregulation in cancer development

The involvement of Telomere Repeat-binding Factors 1 and 2 (TRF1 and TRF2) in cancer development underscores the delicate balance these proteins maintain in cellular homeostasis. As key components of the shelterin complex, TRF1 and TRF2 are essential for telomere integrity and chromosome stability. Their dysregulation, encompassing both overexpression and underexpression, is closely linked to oncogenesis. In the perspective of cancer, TRF1 contribution is complex. Normally, it regulates telomere length and assists in telomere replication. However, in various cancers, including lung and breast cancers, TRF1 is often overexpressed, leading to abnormally long telomeres, which can drive genomic instability and foster tumor development (Muñoz et al. 2005). On the other side, reduced TRF1 expression is associated with telomere shortening and chromosomal instability, a common feature in cancer cells (Martínez and Blasco 2010). TRF2’s role in cancer revolves around its capacity to shield telomeres from being identified as sites of DNA damage. In several cancers, including melanoma and glioblastoma, TRF2 is typically overexpressed. This helps cancer cells maintain telomere integrity, despite critically short telomeres, facilitating continued proliferation and evading replicative senescence. Additionally, TRF2 overexpression is linked to increased telomerase activity, a trait of many cancer cells, aiding their immortalization (Karlseder et al. 1999b; Khodadadi et al. 2021). Conversely, TRF2 downregulation or loss leads to telomere deprotection and genomic instability, fueling early cancer development. The absence of TRF2 function can cause chromosome fusions and initiate genomic rearrangements through breakage-fusion-bridge (BFB) cycles, a potent driver of cancer (Loayza and de Lange 2003). The synergistic effects of TRF1 and TRF2 dysregulation in cancer further complicate their roles. Alterations in their expression can disrupt telomere length balance, resulting in either elongation or excessive shortening, both conducive to genomic instability and cancer progression (Martínez and Blasco 2010). Additionally, the impact of TRF1 and TRF2 extends beyond telomere length regulation to include modulation of DNA damage responses, apoptosis, and cell cycle control, all crucial aspects in cancer biology (Smogorzewska and de Lange 2002). The potential for targeting TRF1 and TRF2 in cancer treatment is a promising research area. Their pivotal roles in telomere maintenance and the unique telomere dynamics in cancer cells make them attractive therapeutic targets. Developing strategies to manipulate their activity or disrupt their interaction with telomeres could offer new avenues in cancer therapy, exploiting the specific vulnerabilities of cancer cell telomeres (Blackburn et al. 2015).

### Structural organisation of TRF1 and TRF2

TRF1 and TRF2 share a similar molecular architecture, characterized by a C-terminal Myb/SANT DNA-binding domain and an N-terminal TRFH domain (Fairall et al. 2001). The crystal structure of the TRFH domains of TRF1 and TRF2 reveals that the heterodimerization of TRF1 and TRF2 is impeded by crucial amino acid differences in the main dimerization interface and TRF1/TRF2 heterodimers are not formed in vitro or in vivo (Ye et al. 2004a). TRF1 and TRF2 directly bind double-stranded telomeric DNA through their Myb/SANT domains and interact with various proteins in the stheltrin complex and asoociated signalling mechanisms to maintain telomere length and structure (Liú et al. 2004). The TRFH domain is essential for dimerization, DNA binding, and telomere localization of TRF1 (Fairall et al. 2001) Fig. 2. TRF2, a distant homologue of TRF1, also binds to telomeric DNA as a homodimer through the Myb-like domain (Wu et al. 2008). The TRFH domain of TRF2 recognizes the TAGGG sequence in the major groove of DNA, with the N-terminal arm locating in the minor groove, similar to TRF1 (Hanaoka et al. 2005). Despite the high degree of structural similarity, there are differences between TIN2 TRFH and the TRFH domains of TRF1 and TRF2 (Hu et al. 2017). TRF2-DNA binding domain is 56% identical to TRF1; however, the loss of TRF2 binding to telomeres has different cellular consequences compared with TRF1 loss (Opresko et al. 2005). TRF2 has been characterized to bind to telomeric double-stranded DNA (dsDNA) as a large oligomeric structure, whereas TRF1 does not exhibit the same degree of oligomerization (Bower and Griffith 2014). The basic N-terminal Gly/Arg-rich (GAR) domain of TRF2 can bind TERRA, but the structural basis and significance of this interaction remain poorly understood (Mei et al. 2021). TRF2 induces chromatin compaction and alters chromatin structure through its Myb/SANT DNA binding domain (Baker et al. 2009, 2011). TRF2 is required for the repression of ATM-dependent signaling and classical nonhomologous end joining (c-NHEJ), whereas TRF1 prevents replication fork stalling in telomeric DNA and represses a fragile site phenotype at telomeres (Frescas and Lange 2014a).

**Fig. 2:**
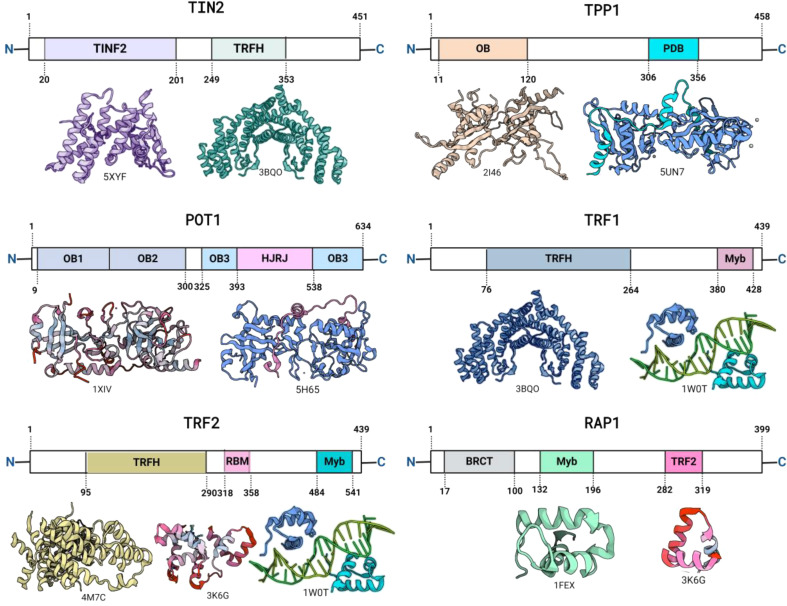
The structural organization of the human shelterin complex, incorporating data from various available structures from PDB with respective PDB structures

The interaction between TIN2 and TRF1/TRF2 is crucial for the stability and function of the shelterin complex at telomeres. TIN2 has been shown to bind to TRF1 and TRF2 simultaneously, stabilizing the TRF2 complex on telomeres (Ye et al. 2004b). Additionally, TIN2 facilitates TRF2-mediated trans- and cis-interactions on telomeric DNA, highlighting its role as an architectural protein in maintaining telomere structure and function (Kaur et al. 2021). Furthermore, TIN2, when tethered to TRF2, can mediate telomere protection by TPP1/POT1, emphasizing its significance in telomere maintenance and protection (Frescas and de Lange 2014b). TIN2 also mediates the functions of TRF2 at human telomeres, indicating its essential role in coordinating the activities of shelterin proteins at telomeres (Kim et al. 2004). To block the interaction between TIN2 and TRF1/TRF2, it is essential to understand the critical residues and domains involved in their binding. Further work is required to elucidate the specific residues critical for TIN2 interaction (Lim et al. 2017). Additionally, the disruption of the shelterin complex by specific molecules may lead to the blocking of TRF1-TIN2 interaction, resulting in cellular senescence (Brankiewicz et al. 2023) Fig. 2. Understanding the molecular basis and quantitative assessment of TRF1 and TRF2 protein interactions with TIN2 is crucial for developing strategies to block their interaction (Kalathiya et al. 2017). Moreover, it has been shown that TIN2 blocks the modification of TRF1 by Tankyrase 1 in vitro, indicating a potential avenue for disrupting the TIN2-TRF1 interaction (Pan et al. 2020).

### Molecular mechanisms of TRF1 and TRF2 in cancer progression

The molecular mechanisms of Telomere Repeat-binding Factors 1 and 2 (TRF1 and TRF2) are pivotal in understanding their roles in cancer progression. These proteins, integral to the shelterin complex, interact with various telomeric proteins and play essential roles in the DNA damage response, contributing to the complexity of cancer biology. TRF1’s function in cancer progression is mediated through its role in telomere length regulation and replication. TRF1 binds to double-stranded telomeric DNA and negatively regulates telomerase access to telomeres, thereby controlling telomere length (van Steensel and de Lange 1997b). Overexpression of TRF1 in certain cancers leads to unusually long telomeres, promoting chromosomal instability and tumor progression (Martínez and Blasco 2010). Furthermore, TRF1 interacts with other telomeric proteins like TIN2, which stabilizes the shelterin complex and contributes to telomere maintenance (Takai et al. 2011). Disruption in these interactions can lead to telomere dysfunction and further facilitate cancer progression. TRF2’s role in cancer is closely linked to its ability to protect telomeres from being recognized as sites of DNA damage. TRF2 forms a protective cap at the end of telomeres, preventing activation of the DNA damage response pathway (Karlseder et al. 1999b; Fan et al. 2021). This mechanism is crucial for telomere stability and preventing end-to-end chromosome fusions. In cancer, TRF2 overexpression helps maintain telomere integrity even in cells with critically short telomeres, allowing cancer cells to evade apoptosis and continue proliferating. Additionally, TRF2 is involved in the repair of double-strand breaks through homologous recombination, a process that is often dysregulated in cancer cells. While the precise role of TRF2 in this process is still debated, studies have demonstrated its recruitment to DNA damage sites to aid in DSB repair (Zhang et al. 2006; Bian et al. 2019). The interplay between TRF1 and TRF2 in cancer progression is also significant. Both proteins are involved in the formation and stabilization of t-loop structures at telomere ends, which are essential for protecting chromosome ends from being recognized as sites of DNA damage (Griffith et al. 1999). In cancer cells, the disruption of these structures can lead to telomere deprotection, genomic instability, and further promotion of oncogenic pathways. Also, TRF1 and TRF2 interact with several other telomeric proteins, including RAP1, TPP1, and POT1, forming a complex network that regulates telomere length and protects telomere integrity (Lange 2005b). These interactions are crucial for the proper functioning of telomeres. In cancer, alterations in these interactions can lead to dysregulation of telomere length and structure, contributing to the cancer phenotype. Another aspect of TRF1 and TRF2’s role in cancer involves the modulation of the DNA damage response (DDR). Normally, these proteins help shield telomeres from the DDR machinery. However, in cancer cells, the DDR at telomeres can be aberrantly activated due to telomere shortening or dysfunction. This aberrant DDR can lead to cell cycle arrest, apoptosis or in some cases, genomic instability and tumorigenesis (Denchi and de Lange 2007). The downstream effects of TRF1 and TRF2 dysregulation in cancer also involve alterations in the expression of genes involved in cell proliferation, DNA repair, and apoptosis. For instance, TRF2 overexpression has been associated with the upregulation of pro-survival pathways and downregulation of apoptotic pathways, providing a survival advantage to cancer cells (Okamoto et al. 2013).

### Targeting TRF1 and TRF2 in cancer therapy

**Fig. 3:**
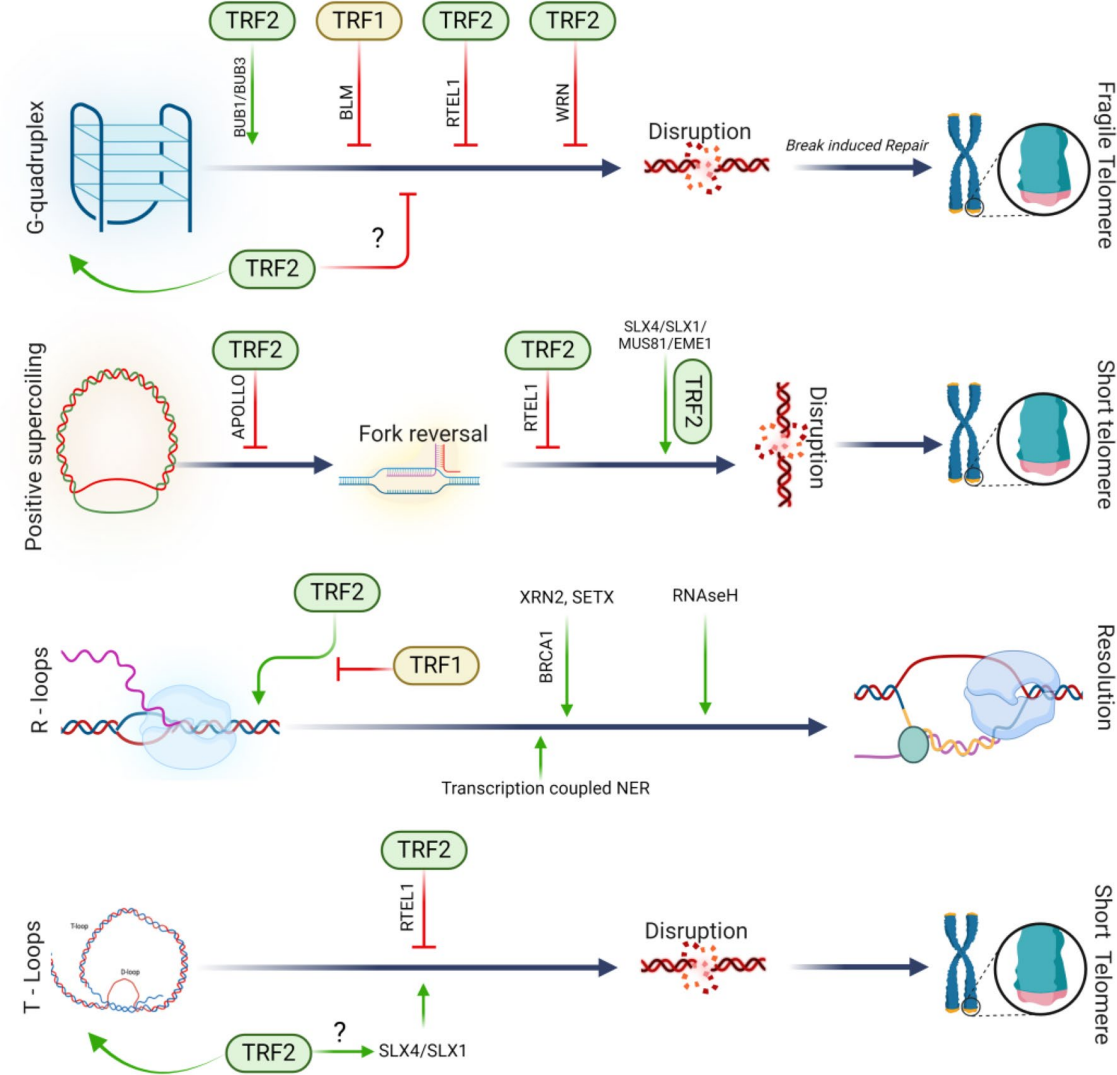
Schematic representation showcasing the diverse mechanisms through which TRF1 and TRF2 exert their influence on telomeric replication, strategically mitigating potential adverse impacts on telomere size or state. The diagram provides a visual depiction of the intricate interplay between TRF1 and TRF2 and their roles in safeguarding the integrity and functionality of telomeres during the replication process

Targeting TRF1 and TRF2 in cancer therapy represents a burgeoning field, with research focusing on the development of novel therapeutic strategies. These strategies include small molecule inhibitors, gene therapy approaches, and combination therapies with other cancer treatments. The rationale for targeting TRF1 and TRF2 lies in their crucial roles in telomere maintenance and the regulation of telomerase activity, both of which are often dysregulated in cancer cells. TRF1 and TRF2 are part of the shelterin complex that protects telomeres and maintains genomic stability. In cancer, aberrations in the functions or expression levels of TRF1 and TRF2 are common and contribute to telomere lengthening or shortening, leading to chromosomal instability (Blackburn 2005). Therefore, disrupting the functions of TRF1 and TRF2 in cancer cells presents a strategic approach to hinder their proliferation and induce apoptosis. One area of focus has been the development of small molecule inhibitors targeting TRF1 and TRF2. These inhibitors are designed to disrupt the binding of TRF1 and TRF2 to telomeres, leading to telomere uncapping and triggering DNA damage responses in cancer cells. For instance, a study conducted in *Saccharomyces cerevisiae* by Smith et al. (Smith et al. 2011) demonstrated that small molecule inhibitors targeting the TRF1-DNA interface could destabilize telomere structure, leading to cell death in cancer cells. Similarly, compounds targeting TRF2, such as G-quadruplex stabilizers, have shown potential in disrupting telomere protection, thereby inhibiting cancer cell growth (De Cian et al. 2008; Ségal-Bendirdjian and Gilson 2008a). Gene therapy approaches have also been explored, focusing on the downregulation or silencing of TRF1 and TRF2 in cancer cells. Techniques such as RNA interference (RNAi) and CRISPR/Cas9 have been employed to specifically target and knock down TRF1 and TRF2 expression. Studies using RNAi against TRF2 have shown that the loss of TRF2 induces telomere deprotection and senescence in cancer cells (Karlseder et al. 2004). CRISPR/Cas9-mediated knockout of TRF1 or TRF2 in cancer cell lines has resulted in reduced proliferation and increased apoptosis (Hu et al. 2012). Combination therapies that include targeting TRF1 and TRF2 along with other cancer treatments have also shown promise. The rationale behind these combination therapies is that targeting the telomere maintenance mechanisms can sensitize cancer cells to conventional therapies such as chemotherapy or radiation. For example, combining TRF1 or TRF2 inhibitors with DNA-damaging agents has been shown to enhance the therapeutic efficacy, leading to increased cancer cell death (Gobbini et al. 2014; Maciejewska et al. 2022). Additionally, the combination of telomere-targeting agents with immune checkpoint inhibitors is an area of active research, with the potential to improve the immune-mediated clearance of cancer cells (Martínez and Blasco 2011). Despite these advancements, there are challenges in targeting TRF1 and TRF2 for cancer therapy. One significant challenge is the specificity of these treatments. Since TRF1 and TRF2 are essential for normal cellular function, their inhibition can also affect normal cells, leading to potential side effects such as increased genomic instability or off-target effects (Palm and de Lange 2008). Therefore, developing strategies that specifically target cancer cells while sparing normal cells is crucial for the successful application of these therapies. In addition, the development of resistance to TRF1 and TRF2-targeted therapies is a concern. As with other targeted cancer therapies, cancer cells can develop resistance mechanisms, such as compensatory upregulation of other telomere-binding proteins or alternative lengthening of telomeres (ALT) pathways (Cesare and Reddel 2010a). Overcoming these resistance mechanisms requires a deeper understanding of telomere biology and the development of novel therapeutic agents or combination strategies Fig. 3.

### TRF1 and TRF2 as a biomarker for cancer diagnosis and prognosis

The exploration of Telomere Repeat-binding Factors 1 and 2 (TRF1 and TRF2) as biomarkers in cancer diagnosis and prognosis is an increasingly significant area in oncological research. These proteins, central to telomere function and integrity, show potential in reflecting the pathological state of various cancers. The analysis of their roles in cancer, particularly focusing on their sensitivity and specificity in diagnostic and prognostic contexts, reveals their potential utility in clinical oncology. TRF1 and TRF2’s involvement in telomere protection and length regulation underpins their relevance in cancer biology. Abnormal expressions of TRF1 and TRF2 are often linked with telomere dysfunction, which is a characteristic feature of many cancer cells. This dysfunction typically manifests as either telomere lengthening or shortening, leading to genomic instability, a common pathway in cancer development (Blackburn 2005). Therefore, the assessment of TRF1 and TRF2 expression could serve as a valuable indicator of oncogenic activity. In cancer diagnostics, the altered expression levels of TRF1 and TRF2 in cancer tissues compared to normal tissues suggest their potential as diagnostic biomarkers. Elevated levels of these proteins have been documented in various cancers, including lung, breast, and colorectal cancers (Smith et al. 2011; Pal et al. 2015). This overexpression is indicative of the cancerous state and may be employed to distinguish cancer cells from normal cells. The prognostic value of TRF1 and TRF2 has been associated with the progression and severity of cancer. High expression levels of these proteins have been correlated with aggressive cancer phenotypes and poorer patient outcomes in several types of cancers. For example, in studies of glioblastoma and hepatocellular carcinoma, increased levels of TRF1 and TRF2 were linked to reduced survival rates and more advanced disease stages (De Cian et al. 2008; Ségal-Bendirdjian and Gilson 2008b). The sensitivity and specificity of TRF1 and TRF2 as biomarkers are crucial for their clinical applicability. Sensitivity refers to the ability to correctly identify cancer patients (true positives), while specificity relates to the correct identification of non-cancer individuals (true negatives). In certain cancers like lung cancer, TRF1 and TRF2 have exhibited high sensitivity, indicating their potential effectiveness in cancer detection (Karlseder et al. 2004). However, the specificity can be variable, and it is influenced by cancer type and other biological factors. Combining TRF1 and TRF2 levels with other established cancer biomarkers could enhance the diagnostic and prognostic accuracy. This approach could provide a more comprehensive view of the cancerous state and assist in devising personalized treatment strategies. Developing reliable assays for measuring TRF1 and TRF2 levels is also essential. Techniques such as immunohistochemistry, ELISA, and quantitative PCR have been employed for this purpose. Refining these techniques to improve accuracy and reliability is an ongoing area of research (Banerjee et al. 2023; Kuan et al. 2023). Despite the promising prospects of TRF1 and TRF2 as cancer biomarkers, challenges persist. Cancer heterogeneity and external factors such as age and lifestyle can influence TRF1 and TRF2 expression, complicating their interpretation. Additionally, ensuring the biomarkers’ specificity and sensitivity across different cancer types and stages remains a critical area of focus.

### Drug discovery efforts and challenges in targeting sheltrin complex

The drug discovery efforts targeting the shelterin complex, a key regulator of telomere function, represent a significant frontier in cancer therapy. This complex, consisting of six core proteins (TRF1, TRF2, RAP1, TIN2, TPP1, and POT1), plays a crucial role in protecting telomeres and maintaining genomic stability. Targeting these proteins offers a novel approach to cancer treatment, but it also presents unique challenges, including drug resistance, off-target effects, and delivery issues (Brankiewicz et al. 2023). The rationale behind targeting the shelterin complex in cancer therapy stems from its central role in telomere protection and the regulation of telomerase activity. Telomere maintenance mechanisms are pivotal in cancer cell immortality, and disruption of the shelterin complex function could lead to telomere uncapping, genomic instability, and subsequent cell death. One approach in targeting the shelterin complex involves the development of small molecule inhibitors that specifically bind to these proteins. Compounds targeting TRF1 and TRF2 have shown promise in preclinical studies (Ivancich et al. 2017; Di Nunno et al. 2023). For instance, inhibitors of TRF1 and TRF2 have been demonstrated to destabilize the shelterin complex, leading to telomere dysfunction and reduced cancer cell proliferation (Smith et al. 2011). These compounds act by disrupting the binding of TRF1 and TRF2 to telomeric DNA, thereby initiating a DNA damage response at telomeres. Another focus area is the development of compounds targeting the interactions between shelterin complex proteins. For example, inhibitors targeting the interaction between TIN2 and TRF1/TRF2 are being explored. These inhibitors aim to disrupt the central scaffolding role of TIN2, thereby destabilizing the entire complex (Kim et al. 1999b; Waksal et al. 2023). Similarly, targeting the TPP1-POT1 interaction is another approach, as this interaction is crucial for POT1-mediated telomere end protection and regulation of telomerase activity (Hockemeyer et al. 2005b). The drug discovery process for targeting the shelterin complex also includes the use of peptide inhibitors. These peptides are designed to mimic the binding domains of shelterin proteins, thus competitively inhibiting their interactions. This approach has the potential to offer high specificity in targeting the complex (Chen et al. 2020). Despite these advancements, developing shelterin-targeted therapies faces significant challenges. One of the primary challenges is drug resistance. Cancer cells can develop resistance mechanisms against these inhibitors, such as compensatory upregulation of other telomere-binding proteins or activation of alternative lengthening of telomeres (ALT) pathways. This resistance diminishes the efficacy of the drugs, necessitating the development of combination therapies or new compounds that can overcome or bypass these resistance mechanisms (Cesare and Reddel 2010b). Given that the shelterin complex proteins are involved in critical cellular functions beyond telomere maintenance, there is a risk that targeting these proteins might affect normal cellular processes, leading to toxicity and side effects. This challenge underscores the need for developing highly specific inhibitors that precisely target the shelterin complex in cancer cells while sparing normal cells. Delivery of these targeted therapies is another hurdle. Ensuring that the drugs effectively reach the tumor site and penetrate the cancer cells to interact with the shelterin complex is critical for their success. The development of efficient drug delivery systems, such as nanoparticle-based carriers, is an area of active research to overcome this challenge.

## Conclusion and perspectives

The exploration of Telomere Repeat-binding Factors 1 and 2 (TRF1 and TRF2), as well as the shelterin complex, represents a significant and evolving area in cancer research and drug discovery. These proteins play crucial roles in telomere maintenance and chromosomal stability, making them key targets in understanding and treating cancer. Primarily, the potential of TRF1 and TRF2 as biomarkers in cancer diagnosis and prognosis is noteworthy. Their altered expression patterns in various cancers compared to normal tissues suggest their utility in early cancer detection and in predicting patient outcomes. While the sensitivity and specificity of TRF1 and TRF2 as diagnostic and prognostic tools show promise, particularly in certain cancer types like lung and breast cancer, the heterogeneity of cancer and influencing factors such as age and lifestyle present challenges in their interpretation and clinical application. In terms of therapeutic strategies, targeting TRF1 and TRF2 in cancer therapy has emerged as a promising approach. Small molecule inhibitors, gene therapy techniques, and combination therapies involving TRF1 and TRF2 disruption have shown potential in preclinical studies. These strategies focus on destabilizing the telomere structure, inducing cell senescence or death, and sensitizing cancer cells to conventional treatments. However, challenges such as drug resistance, specificity, potential side effects, and effective drug delivery need to be addressed for these therapies to be successful in clinical settings. The drug discovery efforts targeting the shelterin complex, comprising TRF1, TRF2, and other components, have also been a significant focus. The development of inhibitors that disrupt the interactions within the shelterin complex or peptide inhibitors mimicking binding domains presents a novel approach in cancer therapy. Yet, the complexity of targeting these proteins without affecting normal cellular functions and overcoming drug resistance and delivery challenges remain significant hurdles. Reflecting on the current state of TRF1 and TRF2 research in the context of cancer and drug discovery, it is evident that significant progress has been made. However, the field is still in its early stages, with much to be understood about the intricate roles of these proteins in cancer biology. Future research should aim to enhance the understanding of TRF1 and TRF2’s roles in different cancer types and stages. This includes elucidating their interactions within the shelterin complex and with other cellular components, their contribution to cancer cell survival and proliferation, and the mechanisms underlying their dysregulation in cancer. Emerging technologies such as CRISPR/Cas9 gene editing, advanced imaging techniques, and high-throughput screening methods will be instrumental in accelerating research in this area. These technologies could enable more precise manipulation of TRF1 and TRF2 and their associated pathways, leading to a better understanding of their functions and interactions. The future prospects for shelterin research are bright, with potential novel therapeutic approaches and new roles of TRF1 and TRF2 in cancer biology. For instance, the development of targeted delivery systems, such as nanoparticle-based carriers, could improve the efficacy and safety of shelterin-targeted therapies. Additionally, the integration of shelterin complex research with immunotherapy, targeted DNA damage response strategies, and personalized medicine could open new avenues in cancer treatment.

## Data Availability

No datasets were generated or analysed during the current study.
